# Antifungal and antitoxin effects of propolis and its nanoemulsion formulation against *Aspergillus flavus* isolated from human sputum and milk powder samples

**DOI:** 10.14202/vetworld.2021.2306-2312

**Published:** 2021-09-03

**Authors:** Alshimaa A. Hassanien, Eman M. Shaker, Eman E. El-Sharkawy, Walaa M. Elsherif

**Affiliations:** 1 Department of Zoonoses, Faculty of Veterinary Medicine, Sohag University, Sohag, 82511, Egypt; 2 Department of Food Hygiene, Faculty of Veterinary Medicine, Sohag University, Sohag, 82511, Egypt; 3 Department of Forensic Medicine and Toxicology, Faculty of Veterinary Medicine, Assiut University, 71515, Egypt; 4 Nanotechnology Research Unit, Animal Health Research Institute, Agriculture Research Centre, 12618, Egypt.

**Keywords:** aflatoxins, *Aspergillus flavus*, mycotoxigenic fungi, propolis nanoemulsion, propolis, Tween 80

## Abstract

**Background and Aim:**

*Aspergillus flavus* causes human and animal diseases through either inhalation of fungal spores or ingestion of mycotoxins as aflatoxins produced in human and animal feed as secondary metabolites. This study was aimed to detect the incidence of *A. flavus* and its aflatoxins in human sputum and milk powder samples and explore the efficacy of pure propolis (PP) and propolis nanoemulsion (PNE) as natural decontaminants against fungal growth and its released aflatoxins.

**Materials and Methods:**

*A. flavus* was isolated by mycological culture and identified macroscopically and microscopically. Coconut agar medium and thin-layer chromatography (TLC) were used to qualitatively detect aflatoxins in the isolated strains. Toxins were extracted from toxigenic strains by the fast extraction technique. The quantitative detection of toxin types was explored by high-performance liquid chromatography (HPLC). PNE was prepared by a novel method using natural components and characterized by Fourier-transform infrared spectroscopy, Zetasizer, and transmission electron microscopy. The effects of PP and PNE on *A. flavus* growth and its toxin were determined by the well-diffusion method and HPLC.

**Results:**

The mycological culture showed that 30.9% and 29.2% of sputum and milk powder samples were positive for *A. flavus*, respectively. TLC confirmed the production of 61.8% and 63.2% aflatoxin by the isolated strains in sputum and milk powder, respectively. PP and PNE showed antifungal activity on *A. flavus* growth with mean±standard error (SE) inhibition zones of 27.55±3.98 and 39.133±5.32 mm, respectively. HPLC revealed positive contamination of toxin extracts with AFB1, AFB2, and AFG2 at 0.57±0.026, 0.28±0.043, and 0.1±0.05 mg/L, respectively. After treatment with PP and PNE, a significant decrease in AFB1, AFB2, and AFG2 concentrations was observed.

**Conclusion:**

This study suggested using propolis and its nanoformulation as antifungal and antitoxins in human medicine and the food industry to increase the food safety level and stop food spoilage.

## Introduction

*Aspergillus* spp. cause human health hazards by either toxin production in food or release of spores in indoor and outdoor environments, such as air, soil, plant, decayed vegetation, and animal feed [1,2]. After *Aspergillus fumigatus*, *Aspergillus flavus* is the second causative agent of invasive and non-invasive aspergillosis [3]. It is responsible for various human diseases, such as hypersensitivity, keratitis, respiratory and cutaneous infections, and invasive aspergillosis in immunocompromised individuals [4].

Several fungal species can grow and survive in dairy products, as they provide numerous substrates, such as proteins, carbohydrates, lipids, and organic acids [5]. Dried milk powder is widely used in Egypt due to the shortage of milk supply; consumers use it as a constituent of yogurt, cheese, bakery products, and hot beverages. The microbiological load of milk products is influenced by the quality of raw milk, production stages, and contamination after heat treatment and storage temperature [6].

Mycotoxins as aflatoxins are secondary metabolites excreted by different fungi, such as *A. flavus* and *Aspergillus parasiticus* in food and/or feed [7]. The most common aflatoxins are AFB1, AFB2, AFG1, and AFG2, with carcinogenic, teratogenic, mutagenic, and immunosuppressive effects. AFB1 is the most prevalent aflatoxin in stored food products [8-10]. Aflatoxigenic fungi are detected by mycological culture aside from thin-layer chromatography (TLC) or high-performance liquid chromatography (HPLC) [11]. Contamination of dairy products by mycotoxins is more attributed to environmental factors that cause fungal growth on these products than ingestion of moldy feed by animals [12]. The main factors of fungal colonization and mycotoxins biosynthesis are changing weather, transportation, and storage of processed food and agriculture crops [13,14]. Different methods have been applied to minimize aflatoxins in animal feed, such as aflatoxin binders, ozone fumigation, essential oils, propolis, and biocontrol of toxigenic fungi, to stop its growth and prohibit mycotoxin excretion [15].

Propolis is a natural bee product that contains different natural compounds, such as vegetable balsam, wax, resin (phenolic acids and flavonoids), and aromatic and essential oils. Its constituents vary according to the geographical distribution of the hive and its botanical origins. Propolis attracted scientific attention due to its biological activities, such as antiviral, antioxidant, antibacterial, anti-inflammatory, antifungal, and anticancer effects [16,17]. Using propolis in human medicine has increased tremendously due to its effectiveness, low side effects, cheap cost, and natural origin [18]. Propolis is also used for food preservation due to its antioxidant and antimicrobial effects, but it is not acceptable by consumers because it has a strong flavor that alters the characteristics of food. Therefore, encapsulation of propolis in nanoemulsion (PNE) between 20 and 200 nm will reduce its effect on the food taste and increase its antimicrobial activity [19,20].

This study aimed to detect the incidence of *A. flavus* and its aflatoxins in human sputum and milk powder, and the toxigenic strains were exposed to pure propolis (PP) and PNE which prepared by a novel method using organic components to investigate their effects on *A. flavus* growth and its aflatoxin levels.

## Materials and Methods

### Ethical approval and Informed consent

The study was approved by the Ethical Committee of Faculty of Veterinary Medicine, Sohag University, Egypt. Participation of the patients was optional and samples were collected after their consent.

### Study period, design, and location

This study was performed from November 2019 to February 2021. Two sample types were selected to detect the incidence of *A. flavus* using mycological examination: Sputum of respiratory patients admitted to Sohag Governmental Hospitals and milk powder sold in markets and groceries in different regions in Sohag City, Egypt. The populations under study constituted patients with respiratory diseases, such as sinusitis, asthma, pneumonia, chronic pulmonary obstructive disease, and chronic bronchitis. Isolated strains of *A. flavus* from patients and food were examined for the presence of toxins by coconut agar medium (CAM) and TLC. The fast extraction technique was used for toxin extraction, and the quantity and types of toxins were determined by HPLC. Toxigenic strains were exposed to PP and PNE to determine their effects on *A. flavus* growth using the well-diffusion method as well as its toxin extract using HPLC.

### Sample collection and mycological examination

A total of 110 sputum samples were collected from respiratory patients admitted to Sohag Governmental Hospitals, and 130 milk powder samples were purchased from several retail markets around Sohag City. Sputum samples were diluted with sterile saline (1:1) and mixed by vortexing [21]. Milk powder samples were prepared by adding 10 g of each sample to 90 ml sterile distilled water and mixed by a homogenizer (Daihan, Korea) for 90 s [22]. All samples (sputum and milk powder) were cultured on potato dextrose agar (PDA) for 5 days at 25°C. Fungal growth was examined for *A. flavus* macroscopically and microscopically at Assiut University Mycological Center.

### Detection of aflatoxins in *A. flavus* strains

*A. flavus* strains were grown in 100 mL malt extract broth for 8 days at 25°C and inoculated on CAM (BD Difco Laboratories, USA) [23]. CAM was observed on days 4 and 8 after inoculation under ultraviolet (UV) light at 245 and 365 nm. The presence of aflatoxins was illustrated by the appearance of a fluorescent halo around the colony. Aflatoxins were then extracted chemically [24].

### Preparation of *A. flavus* aflatoxin extract and TLC screen

The fast extraction technique was used for aflatoxin extraction from CAM according to Moreno *et al*. [25] on days 4 and 8 after inoculation. The obtained aflatoxin in the chloroform extract was analyzed qualitatively by TLC using an external specific aflatoxin stander, UV light at 254 and 365 nm for visualization, and acetone/chloroform (24:176) as a developing solvent.

### Propolis source and component

PP was obtained from the Faculty of Agriculture Farm, Al-Azhar University, Assiut Branch, Assiut, Egypt. Assiut propolis contains different components: Acids, such as lactic acid, hydroxyacetic acid, palmitic acid, 4-methoxy-cinnamic acid, 3,4-di-methoxy-cinnamic acid, isoferulic acid, and caffeic acid; esters, such as 3-methyl 2-butenyl-cis-4-coumarate, 3-methyl 3-butenyl-trans-4-coumarate, 2-methyl 2-butenyl-trans-4-coumarate, 3-methyl 2-butenyl-trans-4-coumarate, isopentenyl caffeate, 2-methyl-2-buteny caffeate, and 3-methyl-2-butenyl caffeate; and flavonoids, such as pinostrobin chalcone, pinocembrin, pinobanksin, and chrysin; and other components, such as glycerol, phosphoric acid, benzyl-2-methyl propyl, 3-hydroxypyridine, and 1,2,3-trihydroxy butanal [26].

### PNE preparation

A novel methodology was used for PNE preparation using only organic material (Tween 80). Propolis powder (200 mg) was added in 100 ml double deionized water containing Tween 80 (3%) with constant stirring for 7 h at 40°C. The solution was sonicated for 10 min and filtered with 200 nm filter.

### Characterization of PNE

PNE was characterized by FTIR for active functional group detection using FTIR spectroscopy (Nicolet, iS10; Thermo Scientific at the Chemistry Laboratory, Faculty of Science, Assiut University), Zetasizer for polydispersity index (PDI), and dynamic nanometer detection (Malvern Analytical, a Spectris Company, UK, at the Nanotechnology Unit, Faculty of Pharmacy, Al Azhar University), and TEM (Jeol, Japan) for morphology and size at the Electron Microscopy Unit in Assiut University.

### Effects of PP and PNE on *A. flavus* growth

*A. flavus* was cultured on PDA for 5-7 days at 25°C. Fungal growth was washed with sterile saline, and the fungal suspension was prepared according to 0.5 MacFarland standards. The fungal suspension (1 mL) was dissolved in Mueller-Hinton agar medium and distributed into Petri dishes, and the wells were made. PP (0.1 g) and PNE (0.1 mL) were added to the wells and left for 48 h. Their effect was evaluated by the diameter of the formed zone around each well [27]. Positive control plates were used without any antifungal compounds.

### Effects of PP and PNE on *A. flavus* aflatoxin

PP and the prepared PNE were added to the aflatoxin extract of *A. flavus* broth (1:1, v/v) and left for 24 h at 25°C. The concentration and type of toxins (AFB1, AFB2, and AFG2) were detected in the control broth (aflatoxin extract only) and treated ones by HPLC (Agilent Technologies 1200 Series, G1321A FLD System, USA). Separation was performed through a Zorbax Eclipse Plus C18 analytical 4.6 × 250 mm, 5 mm column with post-column UVE LC Tech, photochemical post-column, Derivatizer UVC 254 nm, under a temperature set at 30°C. The samples were monitored for UV detection at 365-400 nm and 295 nm excitation and 330 nm emission for fluorescence detection. The samples were eluted with the mobile phase of water/methanol/acetonitrile (55:15:30) at a 1.5 mL/min flow rate, degassed, and filtered with 47 mm × 0.45 mm membrane filter. The injection volume was 30 mL. Peak areas of aflatoxins were recorded and integrated using ChemStation (Agilent). HPLC detection was done at the Analytical Chemistry Unit, Faculty of Science, Assiut University.

### Statistical analysis

Statistical Package for the Social Sciences version 16.0 (IBM, USA) was used to calculate the effects of PP and PNE on fungal growth and aflatoxin content using mean±standard error (SE), analysis of variance, and Tukey’s test. Significant difference was set to p<0.01.

## Results

### Incidence of *A. flavus* and screening of aflatoxins

**Figure-1:**
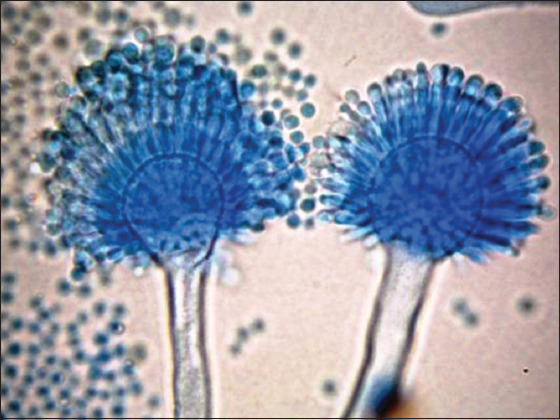
Microscopical examination of *Aspergillus flavus* strains.

**Figure-2:**
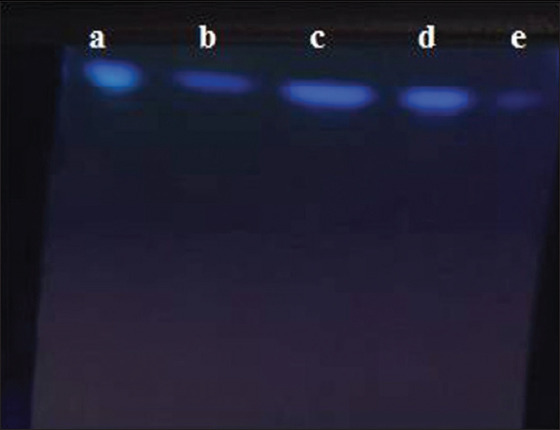
Screen analysis of aflatoxins extract by thin-layer chromatography (TLC) showed positive ultraviolet emission of aflatoxins bands of different sample extracts on TLC plate: a, b: Strains isolated from milk powder; c, d: Strains isolated from sputum; e: Aflatoxin standard.

*A. flavus* was detected in sputum and milk powder samples at 30.9% and 29.2%, respectively (Table-1 and Figure-1). The qualitative detection of aflatoxins by TLC revealed 21 (61.8%) and 24 (63.2%) isolated strains of *A. flavus* from sputum and milk powder, respectively (Figure-2).

**Table 1:** Incidence of *A. flavus* and aflatoxins producing isolates in sputum and milk powder samples.

Samples	*A. flavus* growth		Aflatoxigenic strains	

	No.	%	No.	%
Sputum	34/110	30.9	21/34	61.8
Milk powder	38/130	29.2	24/38	63.2

*A. flavus=Aspergillus flavus*

### Characterization of PNE

**Figure-3:**
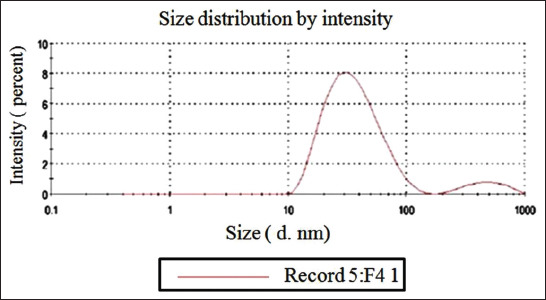
Zetasizer results for propolis nanoemulsion (PNE) showed an average dynamic nanosize± SD (55.95±16.6 nm) and polydispersity index 0.245 of the PNE.

**Figure-4:**
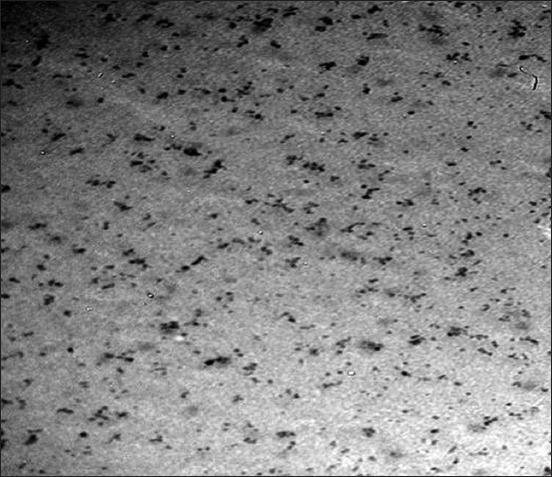
TEM image for propolis nanoemulsion (PNE) showed the PNE with average size of 36.66 nm.

**Figure-5:**
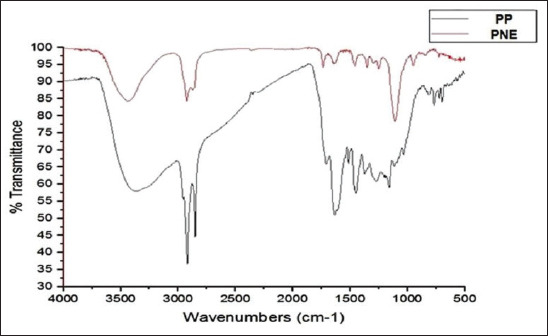
Fourier transform infrared results for pure propolis and propolis nanoemulsion.

Novel modifications in PNE preparation yielded a new nanocompound with specific properties. The average dynamic nanosize by Zetasizer reached 55.95±16.6 nm in diameter. The prepared PNE showed good stability of nanosolutions, and the PDI was 0.245, which was lower than 0.5 (Figure-3). This value represents a stable and dispersed suspension of PNE, that is, there is no tendency to form aggregates in a short period. The TEM image revealed that PNE morphology is nearly spherical, with an average size of 36.66 nm (Figure-4). FTIR analysis revealed that PNE formed new bonds by converting PP to nanopropolis compound (Figure-5).

### Inhibitory effects of PP and PNE on *A. flavus* growth

**Figure-6:**
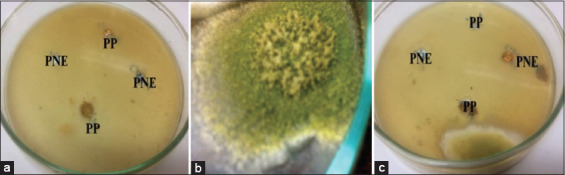
Inhibitory effect of pure propolis (PP) and propolis nanoemulsion (PNE) on the growth of *Aspergillus flavus* isolates: (a) The control potato dextrose agar (PDA) agar medium contains PP and PNE only. (b) Growth of *A. flavus* on PDA without any treatment. (c) Zone of *A. flavus* growth inhibition after treatment with PP and PNE.

PP and PNE have inhibitory effects on *A. flavus* growth (Figure-6), with a mean±SE inhibition zone of 27.55±3.98 and 39.133±5.32 mm, respectively, with a significant difference at p*<*0.01 between PP and PNE (Table-2).

**Table 2:** The efficacy of PP and PNE against *A. flavus* growth on PDA medium.

Fungus type	Inhibition zone (mm)						p-value

	PP			PNE			
	
	Min	Max	Mean±SdE	Min	Max	Mean±SdE	
*A. flavus*	12	39	27.55±3.98	15	45	39.133±5.32	0.01

*A. flavus=Aspergillus flavus*, PP=Pure propolis, PNE=Propolis nanoemulsion, PDA=Potato dextrose agar

### Determination of aflatoxin concentrations before and after treatment with PP and PNE using HPLC

The control samples without any treatment showed a positive detection of AFB1, AFB2, and AFG2 at different concentrations of 0.57±0.026, 0.28±0.043, and 0.1±0.05 mg/L, respectively. Treatment of the positive extracts with PP and PNE showed a detoxification effect with significant levels, and PNE had a significant difference from PP at AFB2 (Table-3).

**Table 3:** Detoxification impact of PP and PNE on aflatoxin extracts using HPLC.

Type of aflatoxin (mg/L)	Control	PP	PNE
		
	Mean±SdE	Mean±SdE	Mean±SdE
AFB1	0.57±0.026	0.37±0.046*	0.24±0.003*
AFB2	0.28±0.043	0.16±0.03*	0.05±0.0*a
AFG2	0.1±0.05	0.06±0.002*	0.04±0.001*

* Significant difference with control when comparison at p<0.01. ^a^Significant difference between the effect of PP and PNE when compared at p<0.01. PP=Pure propolis, PNE=Propolis nanoemulsion, HPLC=High-performance liquid chromatography

## Discussion

### Incidence of *A. flavus*

*A. flavus* produce spores with an average size of 3-6 mm that spread in air and soil. Spores can resist the extreme conditions of the atmosphere due to their hydrophobic nature. Adhesion of conidia to lung cells coupled with macrophage dysfunction, especially in immunocompromised patients, is the initial step in pulmonary aspergillosis [28].

*A. flavus* was detected by mycological culture in 30.9% of the respiratory patients (Table-1). In contrast, Tashiro *et al*. [29] and Sharma *et al*. [30] isolated *A. flavus* in 5% and 7%, respectively. This diversity may be related to the fungal distribution in the environment, which varies according to the geographical distribution and climate, antifungal resistance, and pathogenic potential [4]. Early presentation is non-specific or silent, and the clinical symptoms, prognosis, and infection rate depend on the immunocompromised host. Therefore, timely treatment is important for patient survival [31].

*A. flavus* is the most common fungal species producing aflatoxins during food storage. The factors that enhance *A. flavus* growth and aflatoxin production are temperature, pH, storage time, light, carbon and oxygen content, and thermal/mechanical damage [32,33].

The mycological culture of milk powder showed that 38 of 130 (29.2%) samples were positive for *A. flavus* (Table-1). Higher results were reported by Hassan and Hammad [34] who identified *A. flavus* in milk powder at 60%. Fungal contamination of milk powder may occur during its production stages, packaging, and transportation. Furthermore, improper storage enhances fungal growth and mycotoxin production [35].

### Characterization of PNE

PNE preparation using organic Tween 80 makes irreversible micelles (oil/water emulsion), with a significant decrease in nanopropolis preparation time compared to the previous studies that reported an average time from overnight to 1 day [36,37]. Using ethanol as a dissolvent for propolis is unsafe to use with food and decreases propolis solubility, but this new method is safer and non-toxic and increases propolis solubility by Tween 80 in a short time (7 h). Furthermore, this PNE with a small size reached 36.66 nm with spherical shape by TEM without a significant difference from Zetasizer (Figures-3 and 4). In addition, FTIR showed a newly active bond with the absence of the aromatic group in PNE but present in PP, indicating the absence of the off-flavor present in PP. This determined the appearance of new frequency bands characteristic of the carbonyl group (1900-1600 cm^−1^ -C=O stretching). New peaks in PP intensity began to disappear at 750-1280 cm^−1^, with a strong appearance at 1235-1280, 810-905, and 805-875 cm^−1^ for PNE to methyl band shielding, indicating its high solubility than PP. The spherical shape and increased surface area of PNE are its new properties, increasing its antifungal activity and biological reactivity than PP. PNE is a natural perfect food preservative, masking the propolis off-flavor, and preventing degradation [36].

### Detection of aflatoxins by TLC

Screen analysis of aflatoxin extraction confirmed that 21 (61.8%) *A. flavus* strains isolated from sputum samples and 24 (63.2%) from milk powder strains were aflatoxin producers. *A. flavus* in food can grow under favorable humidity and temperature and secrete its metabolites. Aflatoxin contamination can occur at any point through food chain production, transportation, handling, and storage [38].

### Detection of aflatoxins by HPLC

The selected positive TLC samples were analyzed for aflatoxin production levels using HPLC. The fungal extract revealed positive contamination of AFB1, AFB2, and AFG2 with variable concentrations at 0.57±0.026, 0.28±0.043, and 0.1±0.05 mg/kg, respectively (Table-3). Although *A. flavus* was thought to produce only type B aflatoxins, recent reports demonstrated that it could also produce type G aflatoxins. The detection of the aflatoxin-producing potential of *A. flavus* is an important factor for predicting the severity and incidence of aflatoxin contamination [39,40].

Humans and animals are infected by aflatoxins through consumption of aflatoxin-contaminated food or aflatoxins in animal feed and carried over into milk and its products, such as cheese and milk powder. Aflatoxins inside the body are absorbed through the cell membrane to reach the blood circulation directed to different tissues and organs, such as the liver which considered the target organ [41]. There are two forms of aflatoxicosis: The first is acute primary aflatoxicosis that includes acute liver damage, alteration in digestion, edema, and hemorrhage. The second chronic form includes immunosuppressive, teratogenic, mutagenic, and carcinogenic effects [42]. Effective decontamination of mycotoxins should be non-toxic, and food should remain palatable and retain its nutritive value [43].

### Effects of PP and PNE on *A. flavus* growth

PP and PNE were used as natural products against the toxigenic *A. flavus* and their aflatoxin metabolites. PNE was prepared using organic material, such as Tween 80, without the addition of ethanol. The goal was to use this new antifungal material in the food industry and human therapeutics, as it was prepared by organic material only. The results revealed that PP and PNE affect *A. flavus* growth (Table-2 and Figure-6), consistent with Alamyel *et al*. [44], who showed that propolis has an inhibitory effect on *A. flavus*.

### Effects of PP and PNE on *A. flavus* aflatoxins

Treatment of toxin extracts with PP revealed a significant decrease in AFB1, AFB2, and AFG2 concentrations compared to control. The toxin extracts treated with synthesized PNE showed a significant difference in all toxin concentrations compared to control, and PNE had a significant difference from PP in AFB2 detoxification (Table-3). Similar results were recorded by Abdelazim *et al*. [15], who mentioned that propolis has an inhibitory effect on aflatoxins G1 and B1.

## Conclusion

This study detected the incidence of *A. flavus* in human sputum and milk powder and demonstrated the inhibitory effects of PP and PNE on toxigenic *A. flavus* growth and aflatoxins *in* *vitro*. A novel methodology with less expenses for nanoemulsion formulation of propolis with new properties was identified in this study. Because these results are under laboratory-controlled conditions, further research should be performed on the *in* *vivo* protocol in different fields, such as human therapeutics and agriculture, to control fungal growth and aflatoxin production to assure food safety.

## Authors’ Contributions

AAH and EMS: Conducted the idea and study design, collection of samples, and literature search. AAH, EMS, EEE, and WME: Performed the laboratory work. AAH and EEE: Data analysis. AAH: Manuscript writing. All authors revised and approved the final manuscript.
